# Effectiveness of interventions to prevent abuse in people living with dementia in community settings: A systematic review

**DOI:** 10.1177/14713012241260476

**Published:** 2024-06-20

**Authors:** Seelia Peter A, M Mukhyaprana Prabhu, Debbie Tolson, Baby S Nayak, Rajeshkrishna P Bhandary, Binil V, Elsa Sanatombi Devi

**Affiliations:** Department of Medical Surgical Nursing, Manipal College of Nursing, Manipal Academy of Higher Education, Manipal, Karnataka,India 576104; Department of General Medicine, 29224 Kasturba Medical College, Manipal Academy of Higher Education, Manipal, Karnataka, India 576104; Alzheimer’s Scotland Centre for Policy and Practice, 6413 University of West of Scotland, UK; School of Health and Life Sciences, 6413 University of West of Scotland, UK; Department of Child Health Nursing, Manipal College of Nursing, Manipal Academy of Higher Education, Manipal, Karnataka, India 576104; Department of Psychiatry, 29224 Kasturba Medical College, Manipal Academy of Higher Education, Manipal, Karnataka, India 576104; Department of Psychiatric (Mental Health) Nursing, Manipal College of Nursing, Manipal Academy of Higher Education, Manipal, Karnataka, India 576104; Department of Medical Surgical Nursing, Manipal College of Nursing, Manipal Academy of Higher Education, Manipal, Karnataka, India 576104

**Keywords:** elder abuse, people living with dementia, interventions, prevention, community setting, systematic review

## Abstract

The abuse of older adults is a societal, and legal issue. A person living with dementia is at high risk for abuse due to their physical and cognitive decline.

**Objectives:**

This review examined the evidence for interventions to prevent the abuse of people living with dementia in the community.

**Methods:**

The articles were retrieved from 2000 to 2023 from six databases, including MEDLINE via PubMed, CINAHL Plus via EBSCO, EMBASE, ProQuest Medical Library, Web of Science, and Scopus. The research articles that focused on finding the effectiveness of interventions for preventing abuse of people living with dementia in community settings were included in this review. The review included randomized controlled trials and pre-test post-test trials only. The quality appraisal of the eligible studies was done using ROB 2 and ROBINS II. The findings were tabulated and narratively synthesised.

**Results:**

Out of 1831 articles, only three were included in this review. Only two RCTs were included in this efficacy review. Both the studies showed that the interventions were not effective in reducing abuse. The studies utilised family caregiver interventions like psychological interventions and online supportive education. The review identified psychological interventions with some evidence. Another study was a quasi-experimental study that used dialectical behaviour therapy as an intervention to reduce abuse occurrence. The study showed low evidence and focused only on reporting of elder abuse as an outcome.

**Conclusion:**

This review found very few studies and was not able to draw a conclusion on the effectiveness of interventions for abuse in people living with dementia. Given the paucity of research, there is a clear need to identify how to overcome the challenges faced in elder abuse research and further refine the development of approaches to reduce elder abuse among people living with dementia in community settings.

## Introduction

The abuse of older adults is a significant issue in the fields of public health, criminal justice, and human rights. According to World Health Organization reports, approximately 17% of older adults report having been abused at home (WHO, 2022a, 2022b). A recent estimate of the prevalence of dementia predicts that by 2050, there would be 152 million cases globally, up from 57 million cases in 2019 (Nichols et al., 2022). With the growing number of older adults, the coming decades will face an exponential increase in the number of people diagnosed with dementia, and the rates of dementia related abuse is expected to increase (Cooper et al., 2009; Dong, Chen, & Simon, 2014, Dong, Simon, et al., 2014; WHO, 2022a, 2022b).

The caregiving challenges of a person with dementia progressively increase in intensity and are known to be demanding. When compared to older persons without dementia, those with dementia are more susceptible to abuse (McCausland et al., 2016), owing to a combination of vulnerabilities such as cognitive impairment, social isolation, communication difficulties, and reliance on caregivers (Fang & Yan, 2018; Tronetti, 2014).

According to a definition formulated by the United Kingdom’s Action on Elder Abuse and recognized by the International Network for the Prevention of Abuse, “Elder abuse is a single, repeated act, or lack of appropriate action, occurring within any relationship where there is an expectation of trust and causing harm or distress to an older person” (WHO, 2008). The subtypes of abuse are classified into psychological or emotional abuse, physical abuse, sexual abuse, financial abuse, and neglect (Hall et al., 2016; WHO, 2022a, 2022b).

Elder abuse has a prevalence rate of 15.7% across 28 countries, according to a meta-analysis (Yon et al., 2017). The types of abuse that are most common are psychological abuse (11.65%), financial abuse (6.8%), neglect (4.8%), physical abuse (2.65%), and sexual abuse (Fang & Yan, 2018).

The multifactorial risk factors for abuse are related to the characteristics of the caregiver, the person living with dementia, their relationship, and the environment (Wiglesworth et al., 2010). Behavioural and psychological problems in people living with dementia, such as repeated questioning, wandering, agitation, and aggression, can trigger caregiver violence. Various studies have reported that significant perpetrator factors, such as caregiving overload (Lee & Kolomer, 2005; Weerd & Paveza, 2005), functional decline, social isolation, substance abuse, depression, stress, and anxiety (Dong & Simon, 2013; Pillemer et al., 2016), contribute to abusive behaviours. The victim risk factors include functional dependence or disability, physical and mental health problems, age, gender, and ethnicity (Pillemer et al., 2016). One finding that linked abuse in people living with dementia was the quality of the relationship that existed in the past and present between the carer and the patient (Cooney et al., 2006). Ageism is one of the societal risk factors for abuse (Pillemer et al., 2016), which warrants political attention.

The abuse of older adults has been a relatively hidden issue and taboo. The perpetuator of abuse among older adults is a person living with them at home in most cases, which includes spouses, adult children, daughter-in-law, paid caregivers, or grandchildren. Even neighbours, friends, relatives, or others who visit older adults at home are also perpetuators of abuse (Acierno et al., 2010; Jackson, 2016).

Abuse has detrimental effects on older individuals’ mental and physical health, causing increased risk of mortality, morbidity, psychological distress, institutionalization, hospital admission, and financial loss (Dong & Simon, 2013; Yunus et al., 2017). These consequences will be even worse for people living with dementia due to cognitive deficits and physical dependency (Dong, Chen, & Simon, 2014, Dong, Simon, et al., 2014; Gimeno et al., 2021; Kaspiew et al., 2016).

The mechanism to identify abuse in aged people, both with and without dementia is not yet defined, and neither are the interventions. It can be quite difficult to choose and assess preventative strategies, especially for people living with dementia (Pillemer et al., 2016). Several steps are suggested around the world to prevent or stop elder abuse, but there is a lack of empirical evidence on what works best.

At the community level, the interventions that aim at prevention, fall into three categories: primary (avoidance of abuse before it starts, focusing on entire populations), secondary (targeting at-risk individuals to mitigate or prevent abuse from developing further), and tertiary (avoidance of abuse by focusing on known perpetrators) prevention (AbdulRaheem, 2023). The WHO suggests a few of them: public and professional awareness campaigns, measures to combat ageism, screening (at-risk victims and abusers), caregiver support programs, stress management, school-based intergenerational programs, respite care, residential care policies, and caregiver education on dementia as measures for primary prevention adopted predominantly in high-income countries (WHO, 2022a, 2022b). Secondary prevention includes interventions that target mandatory reporting of abuse, emergency shelters, self-help groups, programs to improve the mental health for abusers, helplines to provide information, referrals, and support the caregivers. The abuse of older adults can be decreased through the efforts of interdisciplinary teams, including social welfare, education, and the health sector (WHO, 2022a, 2022b). The strategies for preventing, identifying, and responding to maltreatment of older adults can be targeted at people living with dementia, formal and informal caregivers, perpetrators in general, the public, and other stakeholders at the governmental and policy level. There is currently minimal evidence supporting the effectiveness of most of these interventions (WHO, 2022a, 2022b).

An increasing number of systematic reviews focusing on interventions for abuse among older people (Ayalon et al., 2016; Baker et al., 2016; Dong, 2015; Fearing et al., 2017; Mydin et al., 2021; Ploeg et al., 2009; Shen et al., 2021), have been conducted in the literature during the last two decades. Most of the studies clearly state the lack of research evidence for interventions to prevent abuse among older adults. It is noteworthy that none of the reviews were done exclusively for people living with dementia as a beneficiary group, except for one review (Mileski, 2019) that focused on the prevention of abuse in people living with dementia in institutional settings (Mileski, 2019).

According to a comprehensive review of community-based strategies for preventing elder abuse, just one study in a systematic review showed evidence that carers of people living with dementia could benefit from psychological intervention (Fearing et al., 2017). This review focused on all interventions for abuse for older adults in general, irrespective of any specific diagnosis or physical disabilities (Fearing et al., 2017). However, the systematic review by Fearing et al. (2017) focused on studies published up to 2017. In order to determine whether the effects of therapies differ depending on cognitive deficiency, the current review was conducted specifically to investigate interventions for abuse in people living with dementia residing in community settings. The current systematic review was undertaken to identify any further developments in the prevention of abuse among people living with dementia as abuse intervention research has gained momentum in last few years.

Due to the complexity of the problem and ethical issues in conducting research in this area, research on elder abuse interventions have received little attention (Jackson & Hafemeister, 2013; Ploeg et al., 2009; Teresi et al., 2016; Wang et al., 2015). The study done on effects of psychological interventions on abuse by caregivers (Cooper et al., 2016) points out that the findings of intervention research on abuse can be influenced by the fact that the researchers may have to report and intervene when significant abuse occurs. Thus, plan of actions to be taken in such situations should be considered in the study methodology (Cooper et al., 2016).

The causes of abuse are multifaceted (Dong, Chen, & Simon, 2014, Dong, Simon, et al., 2014; Fang et al., 2019; Hancock & Pillemer, 2023; Pillemer et al., 2016; Storey, 2020) and research attention has focused on understanding the nature of the problem (Pillemer et al., 2016; Storey, 2020) interventional research is in its infancy (Bolkan et al., 2023; Burnes et al., 2021; Lachs et al., 2021; Rosen et al., 2019; Teresi et al., 2016).

It is imperative to understand the amount of work done in this area, especially when the interventions for the abuse of older adults are very diverse. This review was initiated and carried out with the goal of determining what works for the prevention of abuse in people living with dementia in community settings, regardless of geographical boundaries. In this review, interventions in community settings refers to any interventions for abuse that targets home-dwelling people living with dementia. The interventions for abuse that are targeted at cognitively intact older adults are not sufficient to cater to the needs of older adults who are cognitively impaired. This brings out an interest in determining what interventions work, particularly for people living with dementia.

## Methods

This efficacy systematic review was guided by Cochrane Handbook of Systematic Review of Interventions and Methodological Expectations of Cochrane Intervention Reviews (MECIR) Standards (Higgins et al., 2023a, 2023b).

The review follows the Preferred Reporting Items for Systematic Reviews and Meta-Analysis (PRISMA) 2020 Checklist (Page et al., 2021). The review protocol was registered and published in PROSPERO (Registration number CRD42021262508).

### Eligibility criteria

The inclusion criteria for eligible studies were as follows: (a) Type of participants: informal caregivers of people living with dementia, such as spouses, family, friends, volunteers, and paid caregivers who are over 18 years old; professionals involved in abuse prevention programs; and people living with Alzheimer’s disease and related dementias (ADRD) aged 60 and above. (b) Type of interventions: any interventions for abuse in community settings (see Supplemental material: Appendix 1) were included in this review. (c) Type of comparators: comparators were any different intervention, no intervention or standard care. (d) Type of outcomes: Primary outcomes were prevention or reduction in any type of abuse (physical, psychological, sexual, financial, and neglect or abandonment); reduction in the occurrence or reoccurrence of abuse; and reduction in potentially abusive behaviours and the secondary outcomes were psychological outcomes of the caregiver, caregiver burden, quality of life and resilience of the caregivers. Peer-reviewed, quantitative studies published in English that had interventions for abuse for people living with dementia in community settings with abuse as an outcome were included. The review included studies carried out in home or community settings, and no exclusion was made based on geographical location. The exclusion criteria for the study were results of trials in conference proceedings and abstracts, non RCTs, feasibility studies, studies with unclear abuse outcomes and those with abuse interventions that are not specifically tailored for abuse prevention in people living with dementia.

### Information sources and search strategy

The databases MEDLINE via PubMed, CINAHL Plus via EBSCO, EMBASE, ProQuest Medical Library, Web of Science, and SCOPUS were searched to find studies on abuse among people living with dementia using the keywords “elder abuse,” “people living with dementia,” “interventions,” and their synonyms. (see Supplemental material: Appendix 1). The key words were tested for accuracy by all authors and experts in the field. The MeSH headings and database taxonomies were the basis upon which the search strategy was developed, together with the Boolean operators “OR” and “AND.” (see Supplemental material: Appendix 2). The search was repeated after adding new phrases based on the synonyms identified by the original searches. Google Scholar was reviewed to obtain an updated list. Again, additional studies were searched based on the reference lists of selected articles, unpublished articles, and grey literature. The grey literature searched included ProQuest dissertations and theses, Open Grey, Google Scholar and websites such as Dementia Australia (https://www.dementia.or.au/), Alzheimer’s Association (https://www.alz.org/) and Alzheimer’s Disease International (https://www.alzint.org/).

The search was performed from March 2022 to October 2023 in various databases, and the articles retrieved were from the years 2000–2023 to determine recent evidence in elder abuse management. Rerun of the searches was performed to identify recent studies that might have been missed in the initial search and found one study (Afshari et al., 2023) which was later added to the review. This review included studies performed in the last 23 years to determine the developments in elder abuse interventions in the last two decades. Ethical approval was not obtained, as this study did not include primary data collection.

### Data collection process and quality assessment

Three subject matter experts (SP, ESD, BV) validated the search technique, and modifications were incorporated while searching all the databases. From the reference lists of the identified papers, a search was performed to find more related articles. The Rayyan Tool for Systematic Literature Reviews was used to screen the articles (Ouzzani et al., 2016). Two separate reviewers (SP, BV) reviewed each paper and abstract independently to ensure that they were appropriate and that any disagreements between the reviewers were settled. When consensus could not be reached, the third reviewer’s (ESD) viewpoint was requested to help resolve the situation. After the title and abstract screening, two reviewers independently performed a full-text review of the articles. Discrepancies were discussed and a final consensus was reached in discussion with the third reviewer.

The methodological quality of the randomised controlled trials was assessed using the Cochrane Collaboration Risk of Bias Tool version 2 (Sterne et al., 2019) and the ROBINS I (Higgins, Thomas, et al., 2023) tool for non-randomised trials. The quality of the articles was evaluated separately by two reviewers (SP, BV), and any discrepancies were resolved through discussion by the third reviewer (ESD).

### Data extraction

Two independent reviewers (SP, BV) collected the pertinent research information from original studies into a validated data extraction form and summary table from the included articles, and then cross-checked the findings. The study details included the name of the first author, year of publication, country, setting, methodology, target population, demographic characteristics, sample size, intervention type, frequency and duration of the intervention, comparison, instruments used and results. The original author of one study was contacted for clarification. The data were checked for accuracy and consistency.

### Data synthesis

The studies varied greatly in terms of study design, interventions and their duration, frequency, mode of delivery, and outcome variables. Two studies were randomised controlled trials and the other study had a pre-post experimental design. Narrative synthesis was performed as the included studies were only two and highly heterogeneous. The findings from the two studies are discussed in terms of study selection, critical appraisal results, characteristics of included studies, details of the intervention, measurement tools, data collection methods and impact of abuse prevention programmes on people living with dementia.

## Results

### Study selection

**Figure 1.**
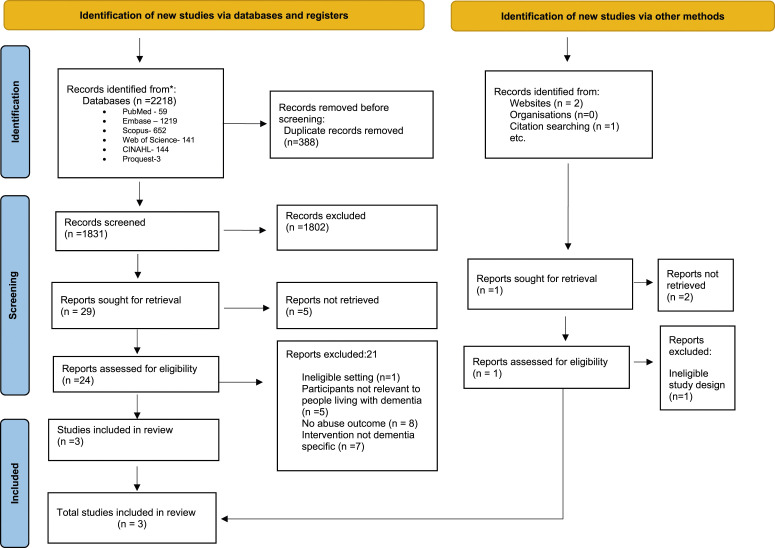
Preferred reporting items for flow diagram. From: Page MJ, McKenzie JE, Bossuyt PM, Boutron I, Hoffmann TC, Mulrow CD et al. The PRISMA 2020 statement: an updated guideline for reporting systematic reviews. BMJ 2021; 372:n71. doi: 10.1136/bmj.n71. For more information, visit: https://www.prisma-statement.org/.

The extraction process of this systematic review is depicted in the PRISMA diagram shown in Figure 1. PRISMA 2020 flow diagram (Page et al., 2021). One thousand eight hundred thirty-one articles were identified after duplicates were removed. Twenty-four articles underwent a full-text review to assess their eligibility in more detail. Twenty-one studies were excluded from this review because they did not meet the inclusion criteria. The study details and the reasons for exclusion are detailed in Table 1.

**Table 1.** Excluded studies.

Author and year	Reason for excluding
Rosen et al., 2022	Ineligible study design
	Ineligible setting.
	Study samples included older adults with and without dementia.
	Intervention was not specific for people living with dementia
Maxwell et al., 2022	Study samples included older adults with and without dementia.
	Intervention was not specific for people living with dementia
Mohd Mydin et al., 2022	Intervention was not specific for people living with dementia
	No elder abuse outcome
Sirey et al., 2021	People living with dementia were excluded in this study.
Oveisi et al., 2021	Ineligible population
Kim et al., 2021	People living with dementia/neurological deficits were excluded in the study.
	No abuse related outcomes
Kunik et al., 2020	No abuse related outcomes
de Oliveira et al., 2019	No abuse related outcomes
Livingston et al., 2020	No abuse related outcome
Gustafson et al., 2019	No abuse related outcomes.
Gaugler et al., 2018	No abuse related outcome.
Estebsari et al., 2018	People living with dementia/neurological deficits were excluded in the study.
Hazrati et al., 2017	People living with dementia were excluded in this study.
Khanlary et al., 2016	The study does not mention whether the samples included caregivers of people living with dementia.
Sirey et al., 2015	Ineligible population
	Ineligible study design
Huang et al., 2013	No abuse related outcomes
Cooper et al., 2012	Ineligible study design
	Intervention is not specific for people living with dementia
Phillips, 2008	Ineligible population
	Intervention is targeted to reduce abuse towards caregivers.
Bartels et al., 2005	Study samples included older adults with and without dementia.
	Intervention was not specific for people living with dementia
Richardson et al., 2002	Ineligible setting
	Intervention was not specific for people living with dementia
Thompson et al., 2000	Ineligible population

Only three studies were included for review as they met all the inclusion criteria. Table 3 shows three citations (Cooper et al., 2016; Livingston et al., 2013, 2014) for a single study. This indicates three follow up articles (2 year follow-up) of a single study. Hereafter mentioned in the text as (Cooper et al., 2016; Livingston et al., 2013, 2014) where ever applicable.

Two of the included articles were randomised controlled trials (Afshari et al., 2023; Cooper et al., 2016; Livingston et al., 2013, 2014), and the other (Drossel et al., 2011) was a non-randomised controlled trial. This review initially focused on randomized controlled trials, but due to a lack of studies, a non-randomized controlled trial was also included later (a deviation from the registered protocol), which resulted in including one more study in the review.

### Critical appraisal results

The three studies included in this review utilized psychological interventions for caregivers of people living with dementia and online supportive education programs for caregivers and reported an abuse outcome. The randomised controlled trial (Cooper et al., 2016; Livingston et al., 2013, 2014) on “START” showed low risk of bias and another randomised controlled trial (Afshari et al., 2023) that investigated the effect of online dementia education on enhancing resilience and preventing abuse of people living with dementia by family carers showed a high risk of bias.

The third study (Drossel et al., 2011) was a pre-post experimental study utilising Dialectical Behaviour Therapy for caregivers which showed very low evidence. The study showed low methodological quality due to its insufficient sample size, lack of control group, and absence of suitable data collection tools to assess abusive behaviours. Table 2 provides the methodological assessment of the included studies.

**Table 2.** Quality assessment of included studies.

a. Quality assessment of RCTs using RoB 2							
Study	Random sequence generation	Allocation concealment	Blinding participants and personnel	Blinding outcome assessment	Incomplete outcome data	Selective reporting	Risk of bias
Livingston et al., 2013	Low	Low	High	Low	Low	Low	Low
Livingston et al., 2014							
Cooper et al., 2016							
Afshari et al., 2023	Some concerns	Some concerns	High	High	High	Low	High

b. Quality assessment of non-randomised studies-of interventions using ROBINS II								
Study reference	Confounding variables	Selection of participants	Classification of interventions	Deviations from intended interventions	Missing data	Measurement outcomes	Selection of reported results	Overall assessment
Drossel et al. 2011	x	x	+	NI	+	x	+	High risk of bias

**Key:** *+, low-risk of bias; -, moderate-risk of bias; X, serious-risk of bias; NI, no information provided*.

### Characteristics of included studies

The study characteristics are summarized in Table 3. One study was conducted in the United Kingdom (Cooper et al., 2016; Livingston et al., 2013, 2014), one in Iran (Afshari et al., 2023) and the other in the United States of America (Drossel et al., 2011). The settings of the studies included mental health trust’s memory services (Cooper et al., 2016; Livingston et al., 2013, 2014), community clinic offering dementia services (Drossel et al., 2011) and participants from a list of active cases of dementia maintained by a non-governmental organization involved in the care of people living with dementia (Afshari et al., 2023).

**Table 3.** Study characteristics.

Study Author, year	Country	Setting	Methodology	Target population facing abusive behaviours	Target population for intervention	Demographic characteristics	Sample size	Intervention type	Frequency and duration of the intervention	Comparison condition	Instruments/measures	Outcome and results
Afshari et al., 2023	Iran	Iran Dementia & Alzheimer’s Disease Association (IDAA)	Randomized controlled trial with follow-up immediately after 2 months of intervention	*N* = 74 people living with dementia.	*N* = 74 Caregivers of people living with dementia.	**Caregivers Intervention group** *Gender* Female: 64.7% Male: 35.3% *Mean Age:* 47.65 ± 9.42 years	*N* = 74 caregivers	Online supportive education program on caregiver’s resilience and abuse which involved a WhatsApp group to share content on dementia, respect and dignity of people living with dementia, vignettes on abuse, techniques to reinforce resilience among caregivers, online consultation by neurologist, stress reduction measures, information on resources available and mutual support from the caregiver group members	2-month intervention period with weekly support sessions and education	Standard care	Caregiver Abuse Screen (CASE)	No significant difference in the scores of abuse (*p* = .447) and resilience (*p* = .08) after the intervention.
			Parallel group Open trial		Allocation **Intervention group:** *N* = 37 **Control Group** N = 37	**Control Group** *Gender* Female: 72.4% Male: 27.6.7% *Mean Age* 46.69 ± 10.11 years					Walsh Family Resilience Questionnaire (WFRQ)	A significant difference was found in the abuse scores before and after the study in the intervention group (*p* = .022) and not for resilience scores.
						**Mean age of people living with dementia** Intervention group 77 ± 7.83 years Control Group 75.46 ± 9.99 years						
Livingston et al., 2013; Livingston et al., 2014; Cooper et al., 2016	UK	London and Essex, United Kingdom Two mental health trusts’ memory services	Randomized controlled trial with long-term follow-up at 4 months, 8 month, 1 yr and 2 yr	*N* = 260 people living with dementia.	*N* = 260 Caregivers of people living with dementia. 2:1 Allocation **START:** *N* = 173	**Caregivers START** Gender: Female: 67.1% Male: 32.9% Mean Age: 62 yrs	*N* = 260 caregivers	The intervention STrAtegies for RelaTives (START) involved a manual based coping intervention, which consisted of psychoeducation about dementia, carers’ stress, emotional support, and behavioural management techniques.	8 manual based individual sessions at home.	Standard care	1. Modified Conflict Tactics Scale 2. The Hospital Anxiety and Depression Scale 3. Zarit Burden Interview	Less abusive behaviour compared TAU group (odds ratio 0.47, 95% confidence interval 0.18 to 1.23) This was not statistically significant. **Follow-up studies** There was no evidence that abusive behaviour levels differed between randomization groups or changed over time. A quarter of carers still reported significant abuse after two years, but those not acting abusively at baseline did not become abusive. **Percentage of participants’ abusive behaviour overtime** 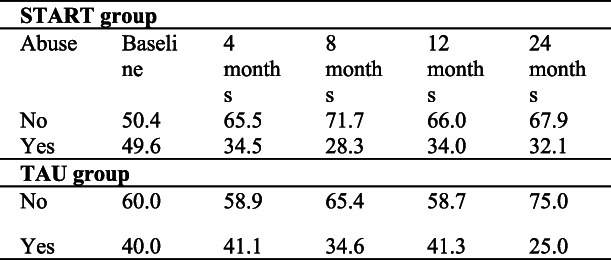 Significant reduction in caregiver burden, decrease in scores on the hospital anxiety and depression scale, at 8,12,24-month follow-up was reported.
			Parallel group single blinded study.		**TAU** N = 87	**TAU:** Gender**:** Female: 71.3% Male: 28.7% Mean Age: 56.1 yrs						
						Mean age of people living with dementia						
						START- 79.9 yrs TAU- 78.0 yrs						
Drossel et al. 2011	USA	State funded community clinics offering dementia services	Pre–post experimental design	Person with Dementia with co-morbidities and having moderate to severe cognitive deficits	Caregivers were referred to DBT Skills by their individual therapists routine clinical setting offering services to individuals with dementia	Caregivers Mean Age: 59 yrs	24 Caregivers of older adults with dementia	Manualized Dialectical Behaviour Therapy Group Sessions	Number of sessions: 8 5 hours of direct contact per week	No comparator	Mandatory Reporting of abuse incidents to authorities. No measurement tools to assess abusive behaviours.	Elder abuse: 2 of 16 caregivers reported elder neglect to authorities.
						Gender: Men- 21% Women 79%	Only 16 completed the intervention				Center for EpidemiologicalStudies-Depresssion Scale (CES-D)	Depression scored improved by 10% among 40% of the samples.
											Caregiver Burden Inventory (CBI), Maslach Burnout Inventory (MBI)	No statistically significant reduction in caregiver burden
											Ways of Coping Checklist (WoC-R)	***Statistically significant*** increase in coping.
											Short Form Health Survey (SF-36)	***Statistically significant*** increase in emotional wellbeing and energy level.

*Note.* STrAtegies for RelaTives (START), TAU- Treatment As Usual.

A total of 358 caregivers of people living with dementia were recruited in the studies included in this review. The number of participants in the DBT study (Drossel et al., 2011) was only 24, and only 16 caregivers completed the intervention. The mean age of the caregivers ranged from 47.65 years to 62 years. Regarding gender, the majority of the caregivers were females in all the studies.

### Details of the intervention

The interventions adopted by all the three included studies were tailored for family caregivers. Two of the included studies (Cooper et al., 2016; Drossel et al., 2011; Livingston et al., 2013, 2014) used psychological intervention for caregivers of people living with dementia to reduce abuse occurrence. Another RCT (Afshari et al., 2023) used online supportive education for family caregivers to reduce abuse.

The number of sessions for START was eight, delivered in home settings on an individual basis. The sessions comprised psychoeducation, dementia education, handling caregiver stress, caring for a person living with dementia, common problems faced by a caregiver, difficult caregiving situations and ways to tackle them, communication techniques, coping strategies, future needs, legal planning, pleasant activities, and reinforcing techniques learned in previous sessions (Cooper et al., 2016; Livingston et al., 2013, 2014).

A study (Afshari et al., 2023) that used online supportive education for caregivers utilized a WhatsApp group to share content on dementia education, respect and dignity of people living with dementia, videos on dementia, vignettes on abuse and neglect and strategies to strengthen resilience among caregivers of people living with dementia. The concerns and challenges faced by caregivers were addressed by one of the research team members and a weekly online consultation by a neurologist was provided (Afshari et al., 2023).

Another study (Drossel et al., 2011), adopted a psychological intervention called Dialectical Behavioural Therapy (DBT). The manual included a step-by-step guide for mindfulness techniques, interpersonal efficiency, ability to tolerate distress, and emotion regulation. DBT was a group therapy that covered four modules delivered over eight weeks. The sessions were delivered by graduate and master’s-level doctoral student therapists specializing in clinical psychology who have undergone DBT training and supervision. The caregivers took part in eight 2.5-h sessions every week. The program was provided at a community clinic, which included telephone booster sessions, a 24-h helpline, and support services.

### Data collection and measurement tools

A total of 173 caregivers were included in the START intervention, and 87 caregivers were in the “treatment as usual” (TAU) group, which received standard care and the participants were not blinded. The START study was a long-term study with follow-ups at 4 months, 8 months (Livingston et al., 2013), and 12 months and 24 months (Cooper et al., 2016; Livingston et al., 2014). The Modified Conflict Tactic Scale (MCTS) was used in the START study to obtain self-reports of potentially abusive behaviours from the family caregivers, where the caregivers must rate how often the mentioned ten abusive behaviours occurred in the last 3 months. The financial abuse and sexual abuse components are not assessed using this tool. The other study (Drossel et al., 2011) did not use any structured tools to assess the abusive behaviours of the caregivers at baseline or at follow-up. Only the mandatory report of abuse to Elder Protective Services was recorded and analysed at baseline and soon after the completion of the sessions.

### Effectiveness of interventions to prevent abuse in people living with dementia

The findings are reported as abuse outcomes, mental health outcomes, caregiver burden, caregiver quality of life and caregiver resilience.

#### Abuse related outcomes

Abuse was the primary outcome in one study (Afshari et al., 2023), and both the other studies (Cooper et al., 2016; Drossel et al., 2011; Livingston et al., 2013, 2014) measured abuse as a secondary outcome. A study (Afshari et al., 2023), which assessed family carer abuse of people living with dementia, found no statistically significant difference in abuse scores (*p* = .447) following the intervention. However, the study reported a noteworthy difference (*p* = .022) in the abuse scores of the intervention group pre- and post-interventions.

In the START study (Cooper et al., 2016; Livingston et al., 2013, 2014), there was a reduction in abusive behaviours, but with no statistical significance. The percentage of family caregivers who reported abusive behaviours was 49.6 at baseline in the START group which was reduced to 28.3% at 8 months. However, at the one-year follow-up, there was an increase in the abuse reports in the START group to 34% and then a reduction to 32.1% by the two-year follow-up. After two years, 25% of carers still reported considerable abuse; however, individuals who had not previously acted abusively did not become abusive (Cooper et al., 2016). However, this study noted that the median abuse scores decreased over two years in both the intervention and control groups.

The second study (Drossel et al., 2011) also examined the impact of DBT on abuse among caregivers of people living with dementia. During the study period, elder neglect by two of 16 caregivers was reported by the therapists to the authorities. Due to the small sample size, further statistical inferences are not mentioned in the study.

### Mental health outcomes

Two included studies had mental health outcomes as the primary outcome. Poor caregiver mental health and caregiver burden are risk factors for the abuse of people living with dementia. The START study (Livingston et al., 2013, 2014) focused on the psychological well-being of the caregiver. The primary outcomes of the START study were caregiver depression and anxiety. The START intervention could reduce caregiver anxiety and depression (Livingston et al., 2013, 2014). The depression and anxiety scores adjusted for baseline showed a mean difference (*p* = .02) in favour of the START intervention.

The DBT study (Drossel et al., 2011) also had mental health outcomes as the primary outcomes which included caregiver depression, caregiver well-being and coping (Drossel et al., 2011). The study revealed a statistically significant increase in coping (*p* < .005), emotional well-being (*p* < .004) and energy levels (*p* < .001) in the group that received DBT intervention (Drossel et al., 2011).

### Caregiver burden and burnout

The caregiver burden is one of the major risk factors for abuse of people living with dementia. Two studies examined the effectiveness of psychological intervention on caregiver burden. The START study (Livingston et al., 2013, 2014) showed significant improvement in the caregiver burden in the intervention group. In contrast, the other study (Drossel et al., 2011) showed no significant effect on caregiver burden and burnout.

### Quality of life

Caregiver quality of life was assessed in only one study (Drossel et al., 2011) which showed no significant difference in QoL scores before and after the intervention. Another study (Livingston et al., 2013, 2014) measured care recipient quality of life and showed no intervention effect.

### Resilience

The resilience of caregivers was measured in a study (Afshari et al., 2023) that found no significant difference in the scores of resilience after the intervention.

## Discussion

This review was conducted to find out the available evidence on abuse interventions for people living with dementia. Three studies on community-based strategies to combat abuse in people living with dementia were included in this systematic review. The START study showed a very low risk of bias and reported that psychological interventions for caregivers of people living with dementia could reduce abusive behaviours, but the results were not statistically significant in the initial (Livingston et al., 2013) and all other follow-ups (Cooper et al., 2016). However, the START study reported significant improvement in caregiver mental health outcomes which is a known risk factor for caregiver abuse.

This review suggests that the individual caregiver psychological interventions provided as part of routine care may have a potential in reducing abusive behaviours. This can be complemented with additional support strategies that may bring about changes in caregiver behaviour. This needs further exploration.

One study with low risk of bias (Cooper et al., 2016; Livingston et al., 2013, 2014) did not show statistically significant outcomes regarding abuse intervention, while the other two studies (Afshari et al., 2023; Drossel et al., 2011) showed high risk of bias might have compromised the reliability of the results. Due to the limited number of included studies and the mixed quality of their methodologies, current review suggests that a definitive conclusion cannot be drawn regarding the effectiveness of abuse intervention. It underpins the importance of further research with well-designed studies to provide more reliable evidence on the efficacy of interventions aimed at addressing abuse.

In the discussion section of the present review, reasons for the very low number of abuse intervention studies, difficulties in conducting abuse intervention research and suggestions to improve the design and research in this area are outlined.

The complexity in conducting abuse research is a reason very few studies are taken up in this area. Abuse research is complex and requires careful planning to carry out because of several factors. Notable ones are, the sensitive nature of the topic, the complexity of abuse types and diverse risk factors, legal issues that arise during the research study, data collection methods, acceptability of the participants, and risk of adverse events. The data collection methods in abuse research should be chosen carefully. The self-reports of abuse by caregivers were assessed in two studies (Afshari et al., 2023; Cooper et al., 2016) whereas mandatory reports of abuse were used in the other study (Drossel et al., 2011). The START study (Cooper et al., 2016; Livingston et al., 2013, 2014) reported all types of abuse except sexual abuse and financial abuse. The study (Afshari et al., 2023) that used an online support program measured potential physical/psychological abuse, and neglect whereas the DBT study (Drossel et al., 2011) reported only neglect. The Modified Conflicts Tactics Scale is the most widely used tool to assess abusive behaviours of the caregivers towards people living with dementia as the self-reports of a person living with dementia may not be reliable in all stages of the disease due to cognitive impairment. The self-reports of the caregivers on abuse may also be affected by the social desirability factor. The Caregiver Abuse Screen was used in a randomized controlled trial (Afshari et al., 2023) with abuse as an outcome. Studies also show that caregivers are willing to express or openly admit that they initiate abusive behaviour and tend to seek help (Cooper et al., 2010; Williamson & Shaffer, 2001). A combination of self-report and observational tools provides more reliable data on abuse that has occurred and on potentially abusive behaviours. The caregiver self-reports, direct observation of the caregiver-care receiver interaction, supported by a physical examination of the person living with dementia for any signs of abuse and in some cases self-reports of the person living with dementia can be adopted if the person is in the mild stage of disease and found feasible.

The included studies (Afshari et al., 2023; Cooper et al., 2016; Drossel et al., 2011; Livingston et al., 2013, 2014), did not explore the relationship quality within the caregiver-care-receiver dyad. It is noteworthy that pre- and post-diagnosis relationship quality, the aggressive and abusive nature of the care recipient prior to dementia diagnosis, emotional closeness, and rewarding relationships (Williamson & Shaffer, 2001), are factors believed to affect abusive behaviours exhibited by caregivers that need further exploration in future studies that help in planning interventions that improve relationship quality and closeness.

Regarding the delivery of the intervention, individual interventions are preferred over group interventions especially for caregivers of people living with dementia. The START intervention used individual format as carers found it difficult to attend group intervention due to caregiving responsibilities at home. Group interventions adopted for DBT (Drossel et al., 2011) had increased dropouts and non-completers as the caregivers found group sessions difficult to attend. Dropout rates can be reduced by having parallel sessions at different times so that caregivers can schedule their work accordingly and provide individual support to barriers that prevent them from attending the sessions (Drossel et al., 2011). It is noteworthy that people living with dementia cannot be left alone at any time of day when carer support interventions are being planned for their welfare (Drossel et al., 2011). Online supportive education (Afshari et al., 2023) was found to have no effect on abuse outcomes. Multicomponent interventions with online and in-person support may help caregivers of people living with dementia.

There was no specific session in the START intervention that addressed elder abuse, its causes and risk factors, the behavioural and psychological symptoms of dementia that can lead to abusive behaviours. Additionally, it lacked sessions on anger management, conflict resolution, situations that trigger abuse, and strategies to prevent it. Future interventions can be made more comprehensive with a special focus on abuse and dementia care throughout the intervention sessions.

The abuse risk varies according to the stage of the disease, as dementia is a progressive disease. This would again affect the credibility of the findings as the groups may become heterogeneous and the results vary widely. A research design and methodology that cater to these considerations would serve better. Stratified block randomization of participants according to the stage of dementia, gender of caregiver, relationship of the caregiver with the person living with dementia and other relevant identified risk of a particular population help improve the rigor of the study. This can be a limitation in all the included studies (Afshari et al., 2023; Cooper et al., 2016; Drossel et al., 2011; Livingston et al., 2013, 2014).

Elder abuse interventions should be culturally sensitive, as people in different geographical locations and cultures will have different perceptions and understanding about abuse of older adults. The intervention provided by a therapist who belongs to the same cultural background tends to be more acceptable and beneficial than that provided by a therapist from a different culture. Caregivers who belong to the Asian continent may understand the term “abuse” and scenarios that trigger abuse differently when compared to people living in the European region. The meaning of “home,” respect for older adults, the culture of living in a joint family, and the practice of not calling elders by their name are all specific to people living in Asian cultures. Understanding this and incorporating culture-specific concerns may increase the acceptability of the intervention and improve the outcomes.

All the studies (Afshari et al., 2023; Cooper et al., 2016; Drossel et al., 2011; Livingston et al., 2013, 2014), included in this review, the interventions were directed towards the primary caregiver or a person identified as a caregiver by their therapist. The other family members were not included in the intervention program. The involvement of other family caregivers in the abuse prevention programs would be beneficial even though the primary caregiver is at more risk of exhibiting abusive behaviours because, at times, they take up the responsibility of caring or even give shared care.

Studies have reported harmful or adverse events such as higher rates of abuse after the implementation of abuse interventions (Davis et al., 2001; Davis & Medina-Ariza, 2001). However, none of the included studies reported any such events. Future elder abuse studies can explore further factors contributing to adverse events and plan elder abuse interventions that are safe for people living with dementia.

The multifactorial nature of elder abuse makes it difficult to understand the link between risk factors and its occurrence (Maxwell et al., 2022). Each type of abuse should be explored separately (Fang et al., 2019; Jackson & Hafemeister, 2011; Wang et al., 2015) as the causal factors vary, and it should be noted that in most instances, different types of abuse co-occur (Fraga Dominguez et al., 2021; Hamby et al., 2016; Williams, Racette, et al., 2017). Further studies are required to underpin what initiates abusive behaviour, and the least studied are the personality traits of the caregiver (Fang et al., 2019, 2021) and their past aggressive and abusive nature and childhood trauma (Jackson & Hafemeister, 2011; Johannesen & LoGiudice, 2013, Storey, 2020). An in-depth understanding of all the contributing factors is very important in designing and undertaking elder abuse intervention research (Burnes et al., 2021; Day et al., 2017; Dong, 2015) with flawless methodology. A single intervention that focuses only on a few risk factors may not be useful in mitigating the problem of elder maltreatment. This brings out the need to plan multifaceted interventions by multidisciplinary teams (Fearing et al., 2017; Lachs et al., 2021; Meyer et al., 2019; Yan et al., 2022) that target risk reduction. Moreover, interventions for abuse should focus more on primary prevention (prevent abuse from occurring) than secondary (prevent further abuse) or tertiary (manage consequences after abuse has occurred) prevention (Asay et al., 2013; Browne & Herbert, 1997; Burnes et al., 2020; Meyer et al., 2019) because abuse causes irreversible physical and psychological damage to the lives of people living with dementia.

People living with dementia face many physical and psychological declines and may not be able to report any abuse. However, in the initial stages of dementia, they may be able to do so. The reporting of any act of abuse may not be possible in the moderate and severe stages of the dementia, which often results in silent suffering. An assessment regarding their ability to give self-reports of abuse should be done. In some instances, the reports of abuse by people with dementia can be cross-checked or validated with self-reports of abuse given by caregivers, which increases the reliability of the findings. The methodological clarity in identifying this should be considered in future research. There is a lack of evidence-based data from rigorously controlled designs, and this problem needs to be addressed with utmost importance to cause no harm to the people living with dementia.

Dementia research commonly involves dyadic interventions for the caregiver and the person living with dementia. Dyadic interventions are found to be effective in reducing the behavioural and psychological symptoms of dementia, which are one of the risk factors for caregiver abuse towards the person living with dementia. There has been no intervention research for abuse among people living with dementia targeting patient-caregiver dyads (Afshari et al., 2023; Cooper et al., 2016; Livingston et al., 2013, 2014) to date. This can be considered a major gap, and studies can be undertaken that directly benefit the dyad (Jackson, 2016).

The meaning of elder abuse differs from country to country in terms of cultural diversities, societal makeup, and legal approaches existing in that country (Ploeg et al., 2009). What is considered abusive in some communities will not be perceived as such in another community (Mercurio & Nyborn, 2007; Tauriac & Scruggs, 2006).

The studies on elder abuse interventions are primarily performed in high-income countries, and none were found in low- and middle-income countries (Baker et al., 2016; Fearing et al., 2017). In Asian countries, there are communities with close family ties (Sung & Dunkle, 2009; Yunus, 2021), and older adults take part in family decisions. The legal regulations regarding financial abuse, especially in cognitively impaired individuals, also vary from country to country, which must be taken into consideration when implementing preventive strategies. The abuse interventions that work for people living in low- and middle-income countries are not very well tapped yet (Ayalon et al., 2016; Baker et al., 2016). This calls for future research to be done in low and middle-income countries, considering the cost effectiveness of interventions (Pillemer et al., 2016).

One of the abuse intervention outcomes is delayed hospitalization or institutionalization which was not explored in this review. However, none of the studies had assessed this as an outcome of an elder abuse intervention among people living with dementia, which future researchers can explore.

The caregivers could decline to participate in a study, due to caregiving responsibilities, lack of time, transportation issues, and lack of respite. The fact that carers are also the abusers themselves may be another factor in their unwillingness to take part in abuse study. This is another area of concern to safeguard people living with dementia. The governmental and policy directives to conduct a home visit, offer carers additional follow-up support, and spot instances of abuse ensure the safety of people living with dementia (Brownell & Wolden, 2003). The social stigma and loss of family name may prevent carers from reporting abuse or from taking part in interventions for abuse. It is very important to name the intervention, advertise it, and present the content and delivery of the intervention for abuse reduction by caregivers in a non-blaming way (Drossel et al., 2011).

There exists a significant knowledge gap in elder abuse research especially in intervention research. The problem of abuse in older adults has gained much importance in the last two decades, and several research studies have been done, especially on the prevalence (Erlingsson, 2007; Sooryanarayana et al., 2013; Williams, Davis, & Acierno, 2017; Yan et al., 2015; Yon et al., 2017), risk factors (Dong, 2015; Johannesen & LoGiudice, 2013; Orfila et al., 2018; Pillemer et al., 2016; Yalçın Gürsoy & Tanriverdi, 2023), and protective factors for abuse (Acierno et al., 2010; Bolkan et al., 2023; Dong, 2015). The systematic reviews performed on elder abuse interventions to date, report a lack of intervention studies of good quality.

### Strengths and limitations

The strength of this systematic review is that, this is the first systematic review to examine the effectiveness of abuse interventions for preventing abuse for people living with dementia in community settings.

This systematic review has a few limitations that should be considered when explaining the findings. Only three studies were included in this review which were highly heterogeneous. This review included only studies that had interventions exclusively for people living with dementia. In this review, two included studies had a high risk of bias due to which no definite conclusions could be made regarding the effectiveness of interventions for abuse among people living with dementia. This review was limited to only six database searches and did not include databases specialized in the field of sociology, psychology, and social work. It is possible that as a result, we may have missed pertinent articles on interventions for abuse and neglect in people living with dementia.

## Conclusion

This review aimed to provide evidence on interventions for the abuse of people living with dementia in community settings. This review revealed that only three studies were done on interventions for abuse in people living with dementia. The scarcity of studies on elder abuse interventions is already established by previous systematic reviews done in this field. The review included only three studies of variable quality. Although psychological interventions for dementia carers have shown some promise, no robust recommendation can be made on this basis to prevent abuse and neglect in patients living with dementia. The future research works of high quality is needed to establish evidence and the interventions must be tailored considering the vulnerability, setting, geographical location, and cultural diversity. It is vital to explore culturally diverse interventions and draw conclusions from what interventions work in different cultural contexts and geographical boundaries, as dementia care is highly culture-specific.

## Supplemental Material

Supplemental Material - Effectiveness of interventions to prevent abuse in people living with dementia in community settings: A systematic review Supplemental Material for Effectiveness of interventions to prevent abuse in people living with dementia in community settings: A systematic review by Seelia Peter A, M Mukhyaprana Prabhu, Debbie Tolson, Baby S Nayak, Rajeshkrishna Bhandary P, Binil Vand Elsa SanatombiDevi in Dementia.

## Data Availability

The data that support the findings of this study are available from the corresponding author upon request.*
