# Supplementary material for: Effectiveness of interventions to prevent abuse in people living with dementia in community settings: A systematic review
Source: Dementia (London). 2024 Jun 20;23(8):1327–53. doi: 10.1177/14713012241260476 (PMC11491043; doi:10.1177/14713012241260476)
Supplement: Supplemental Material - Effectiveness of interventions to prevent abuse in people living with dementia in community settings: A systematic review [file sj-pdf-1-dem-10.1177_14713012241260476.pdf]

Appendix 1: Key words and synonyms

Key words	Synonyms
Older adult living with dementia	“older adults” OR "aged" OR "frail elderly “OR elder* OR senior OR “senior citizen” OR "aging “OR old OR “geriatrics" OR "old age" OR "Alzheimer Disease" OR "Dementia" OR “patients with dementia” OR “people living with dementia” OR PwD OR “individuals with dementia” OR “dementia patients” OR “senile dementia” OR “memory loss” OR “cognitive impairment” OR “Cognitive Disorders” OR “Alzheimer’s Disease and Related Disorders”
Elder abuse	"elder abuse" OR “elder maltreatment” OR “elder mistreatment” OR "family conflict" OR "physical abuse" OR "emotional abuse" OR “psychological abuse” OR “financial abuse” OR “sexual abuse” OR "sexual trauma" OR "sex offenses" OR elder neglect OR abandonment OR exploit* OR "fraud" OR "crime" OR "domestic violence" OR "bullying" OR aggress* OR stigmati*
Interventions	“intervention” OR strategy OR “intervention to reduce abuse” OR “elder abuse prevention” OR “prevention of elder abuse” OR “elder abuse control” OR “violence prevention” OR “elder protection” OR “caregiver education” OR “caregiver training” OR “caregiver support” OR “dementia education” OR “aggression prevention” OR “family caregiver program*” OR “informal caregiver support*” OR "communication skills training" OR “skills training group” OR “psychological intervention” OR “manual based coping strategy” OR "psychotherapy" OR "imagery, psychotherapy" OR "psychotherapy, group” OR "Cognitive Behavioural Therapy" OR psychoeducation OR "behaviour therapy" OR "behaviour modification" OR “dialectical behaviour therapy” OR DBT OR “stress management” OR "counselling" OR "family therapy" OR “mindfulness-based stress reduction” OR "relaxation techniques" OR "mind-body & relaxation techniques" OR "exercise" OR "leisure activities" OR "recreation therapy" OR "music therapy" OR “engagement activities” OR "psychosocial support systems" OR "respite care" OR “day care” OR "home nursing" OR "social support" OR "social welfare" OR "self-help groups" OR "emergency shelter" OR “safe houses” OR “telephonic support” OR "hotlines" OR “telephonic helplines” OR "social work" OR "mandatory reporting" OR “elder rights protection” OR "house calls" OR “home visit” OR “nurse visit” OR “community health" OR “neighbour watch” OR "legal services" OR "patient advocacy" OR "group homes" OR “victim screening” OR "financial management" OR “mass media education” OR “public awareness campaigns” OR “multidisciplinary interventions” OR “interprofessional program” OR “multicomponent intervention” OR “interdisciplinary care” OR “web based interventions” OR "internet-based intervention" OR “family support” OR “family interventions” OR "home-based intervention" OR “social service” OR “public health initiatives” OR “elder care initiatives” OR "intergenerational relations" OR “intergenerational program*” OR "community support" OR "home care services" OR “restraint reduction” OR “aged friendly cities” OR "rehabilitation" OR "crisis intervention" OR “conflict resolution”
Randomized controlled trails	"randomi?ed controlled trial" OR "controlled clinical trial" OR "clinical trial" OR "single-blind method” OR "double blinded" OR "comparative study" OR "random allocation" OR randomization OR randomized OR randomised OR randomisation OR "randomizations" OR "randomi?e"

Appendix 2: Search Strategy

PubMed Search Strategy
(("older adults"[All Fields] OR "Aged"[MeSH Terms] OR "elder*"[All Fields] OR ("senior"[All Fields] OR "seniority"[All Fields] OR "seniors"[All Fields]) OR "senior citizen"[All Fields] OR "Aging"[MeSH Terms] OR "old"[All Fields] OR "Geriatrics"[MeSH Terms] OR "old age"[All Fields] OR "Frail Elderly"[MeSH Terms] OR "Alzheimer Disease"[MeSH Terms] OR "Dementia"[MeSH Terms] OR "patients with dementia"[All Fields] OR "people living with dementia"[All Fields] OR "PwD"[All Fields] OR "dementia patients"[All Fields] OR "senile dementia"[All Fields] OR "memory loss"[All Fields] OR "cognitive impairment"[All Fields] OR "Alzheimer's Disease and Related Disorders"[All Fields]) AND ("Elder Abuse"[MeSH Terms] OR ("elder"[All Fields] AND "abuse"[All Fields]) OR "Elder Abuse"[All Fields] OR ("elder"[All Fields] OR "maltreatment"[All Fields]) OR "elder maltreatment"[All Fields]) OR ("Elder Abuse"[MeSH Terms] OR ("elder"[All Fields] AND "abuse"[All Fields]) OR "Elder Abuse"[All Fields] OR ("elder"[All Fields] AND "mistreatment"[All Fields]) OR "elder mistreatment"[All Fields]) OR "Family Conflict"[MeSH Terms] OR "Physical Abuse"[MeSH Terms] OR "Emotional Abuse"[MeSH Terms] OR "psychological abuse"[All Fields] OR "financial abuse"[All Fields] OR "sexual abuse"[All Fields] OR "Sexual Trauma"[MeSH Terms] OR "Sex Offenses"[MeSH Terms] OR ("elder"[All Fields] AND "neglect"[All Fields]) OR "elder neglect"[All Fields]) OR ("abandon"[All Fields] OR "abandoned"[All Fields] OR "abandoning"[All Fields] OR "abandonment"[All Fields] OR "abandonments"[All Fields] OR "abandons"[All Fields]) OR "exploit*"[All Fields] OR "Fraud"[MeSH Terms] OR "Crime"[MeSH Terms] OR "Domestic Violence"[MeSH Terms] OR "Bullying"[MeSH Terms] OR "aggress*"[All Fields] OR "stigmati*"[All Fields] OR "delayed hospitalization"[All Fields]) AND (((("intervention"[All Fields] OR "interventions"[All Fields] OR "methods"[MeSH Terms] OR "methods"[All Fields] OR "intervention"[All Fields] OR "interventional"[All Fields]) OR "elder abuse prevention"[All Fields] OR "prevention of elder abuse"[All Fields] OR (("Elder Abuse"[MeSH Terms] OR ("elder"[All Fields] AND "abuse"[All Fields]) OR "Elder Abuse"[All Fields]) AND ("controling"[All Fields] OR "controls"[All Fields] OR "prevention and control"[MeSH Subheading] OR ("prevention"[All Fields] AND "control"[All Fields]) OR "prevention and control"[All Fields] OR "violence prevention"[All Fields] OR (("elder s"[All Fields] OR "elders"[All Fields] OR "elder"[All Fields]) AND ("protect"[All Fields] OR "protected"[All Fields] OR "protecting"[All Fields] OR "protection"[All Fields] OR "caregiver education"[All Fields] OR ("caregiver support program"[All Fields] OR "dementia education"[All Fields] OR "aggression prevention program*"[All Fields] OR "family caregiver program*"[All Fields] OR (("caregivers"[MeSH Terms] OR "caregivers"[All Fields] OR ("informal"[All Fields] AND "caregiver"[All Fields]) OR "informal caregiver"[All Fields]) AND "program*"[All Fields]) OR "communication skills training"[All Fields] OR ("Psychotherapy"[MeSH Terms] OR "imagery, psychotherapy"[MeSH Terms] OR "psychotherapy, group"[MeSH Terms] OR "psychotherapy, multiple"[MeSH Terms] OR "Cognitive Behavioral Therapy"[MeSH Terms] OR "Interpersonal Psychotherapy"[MeSH Terms] OR "psychotherapy, brief"[MeSH Terms] OR "Person-Centered Psychotherapy"[MeSH Terms]) OR ("psychoeducation"[All Fields] OR "psychoeducational"[All Fields] OR "psychoeducative"[All Fields]) OR "Psychosocial Support Systems"[MeSH Terms] OR "dependency, psychological"[MeSH Terms] OR "Behavior Therapy"[MeSH Terms] OR "behavior modification"[All Fields] OR "dialectical behaviour therapy"[All Fields] OR "stress management"[All Fields] OR "Counseling"[MeSH Terms] OR "Family Therapy"[MeSH Terms] OR "mindfulness-based stress reduction"[All Fields] OR "Relaxation Techniques"[All Fields] OR "mind body relaxation techniques"[All Fields] OR "Exercise"[MeSH Terms] OR "leisure activities"[MeSH Terms] OR "recreation therapy"[MeSH Terms] OR "Music Therapy"[MeSH Terms] OR "engagement activities"[All Fields] OR "Respite Care"[MeSH Terms] OR "day care"[All Fields] OR "Home Nursing"[MeSH Terms] OR "Social Support"[MeSH Terms] OR "Social Welfare"[MeSH Terms] OR "Self-Help Groups"[MeSH Terms] OR "Emergency Shelter"[MeSH Terms] OR "safe houses"[All Fields] OR "telephonic support"[All Fields] OR "Hotlines"[MeSH Terms] OR (("telephone"[MeSH Terms] OR "telephone"[All Fields] OR "telephones"[All Fields] OR "telephoned"[All Fields] OR "telephonic"[All Fields] OR "telephonically"[All Fields] OR "telephoning"[All Fields]) OR ("helpline"[All Fields] OR "helpline s"[All Fields] OR "helplines"[All Fields])) OR "Social Work"[MeSH Terms] OR "Mandatory Reporting"[MeSH Terms] OR (("elder s"[All Fields] OR "elders"[All Fields] OR "elder"[All Fields]) AND ("right"[All Fields] OR "right

s"[All Fields] OR "rightful"[All Fields] OR "rights"[All Fields]) AND ("protect"[All Fields] OR "protected"[All Fields] OR "protecting"[All Fields] OR "protection"[All Fields] OR "protective"[All Fields] OR "protectively"[All Fields] OR "protectiveness"[All Fields] OR "protectives"[All Fields] OR "protects"[All Fields])) OR "House Calls"[MeSH Terms] OR "home visit"[All Fields] OR "nurse visit"[All Fields] OR "nurses, community health"[MeSH Terms] OR (("neighbor"[All Fields] OR "neighbor s"[All Fields] OR "neighboring"[All Fields] OR "neighbors"[All Fields] OR "neighbour"[All Fields] OR "neighbour s"[All Fields] OR "neighbouring"[All Fields] OR "neighbours"[All Fields]) AND ("watch"[All Fields] OR "watched"[All Fields] OR "watching"[All Fields])) OR "Legal Services"[MeSH Terms] OR "Patient Advocacy"[MeSH Terms] OR "Group Homes"[MeSH Terms] OR (("crime victims"[MeSH Terms] OR ("Crime"[All Fields] AND "victims"[All Fields]) OR "crime victims"[All Fields] OR ("diagnosis"[MeSH Subheading] OR "diagnosis"[All Fields] OR "screening"[All Fields] OR "mass screening"[MeSH Terms] OR ("mass"[All Fields] AND "screening"[All Fields]) OR "mass screening"[All Fields] OR "screen"[All Fields] OR "screenings"[All Fields] OR "screened"[All Fields] OR "screens"[All Fields])) OR "Financial Management"[MeSH Terms] OR "mass media education"[All Fields] OR "public awareness campaigns"[All Fields] OR "multidisciplinary interventions"[All Fields] OR "interprofessional program"[All Fields] OR "multicomponent intervention"[All Fields] OR "interdisciplinary care"[All Fields] OR "Patient Care Team"[MeSH Terms]) AND "web based interventions"[All Fields]) OR "Internet-Based Intervention"[MeSH Terms] OR "family support program*"[All Fields] OR "family interventions"[All Fields] OR "social service"[All Fields] OR "Public Health initiatives"[All Fields] OR (("elder care"[Journal] OR ("elder"[All Fields] AND "care"[All Fields]) OR "elder care"[All Fields]) AND ("initiative"[All Fields] OR "initiatives"[All Fields])) OR "Intergenerational Relations"[MeSH Terms] OR "intergenerational program*"[All Fields] OR "Community Support"[MeSH Terms] OR "Home Care Services"[MeSH Terms] OR "restraint reduction"[All Fields] OR "domiciliary care"[All Fields] OR (("Aged"[MeSH Terms] OR "Aged"[All Fields]) AND ("friendliness"[All Fields] OR "friendly"[All Fields]) AND ("cities"[MeSH Terms] OR "cities"[All Fields] OR "city s"[All Fields])) OR "Rehabilitation"[MeSH Terms] OR "Crisis Intervention"[MeSH Terms] OR "conflict resolution"[All Fields]) AND ("Randomized Controlled Trial"[Publication Type] OR "Controlled Clinical Trial"[Publication Type] OR "clinical trial"[All Fields] OR "single-blind method"[All Fields] OR "double blinded"[All Fields] OR "comparative study"[All Fields] OR ("random allocation"[MeSH Terms] OR ("random"[All Fields] AND "allocation"[All Fields]) OR "random allocation"[All Fields] OR "random"[All Fields] OR "randomization"[All Fields] OR "randomized"[All Fields] OR "randomisation"[All Fields] OR "randomisations"[All Fields] OR "randomise"[All Fields] OR "randomised"[All Fields] OR "randomising"[All Fields] OR "randomizations"[All Fields] OR "randomize"[All Fields] OR "randomizes"[All Fields] OR "randomizing"[All Fields] OR "randomness"[All Fields] OR "randoms"[All Fields]) OR "Non-Randomized Controlled Trials as Topic"[MeSH Terms])) AND (english[Filter])
